# AI-based detection of neutrophil dysplasia: an accessible and sensitive model for MDS diagnosis from peripheral blood

**DOI:** 10.1007/s00277-025-06533-5

**Published:** 2025-08-19

**Authors:** Nicole H. Romano, Christian Ruiz, Pascal Schlaepfer, Stefan Balabanov, Stefan Habringer, Corinne C. Widmer

**Affiliations:** 1 Moonlight AI, Courroux, Jura, Switzerland; 2 Department of Haematology and Laboratory Medicine, University and University Hospital Basel, Petersgraben 4, Basel, 4031 Switzerland; 3 Department of Medical Oncology and Hematology, University Hospital Zurich, Zurich, Switzerland; 4 Department of Hematology, Oncology and Cancer Immunology, Charité - Universitätsmedizin Berlin, corporate member of Freie Universität Berlin, Berlin, Germany; 5 German Cancer Consortium (DKTK), Berlin, Germany

**Keywords:** Myelodysplastic syndrome, Computer vision, Neutrophil classifier, Peripheral blood smears morphology

## Abstract

Myelodysplastic syndrome / neoplasm (MDS) presents a diagnostic challenge due to the need of expert morphological analysis, and the reliance on genomic analysis of collected bone marrow material for the definite diagnosis. This study aimed to facilitate this process by developing a computer vision AI-based model that is capable of diagnosing MDS from images from peripheral blood smears (PBS). We used a cohort of 43,371 neutrophils from 84 MDS and 60 non-MDS samples to train a neutrophil classifier to differentiate between dysplastic and non-dysplastic cells. The model was initially fed with PBS images from patients with prominent MDS (pMDS) and further refined to detect non-prominent MDS (npMDS), i.e., without clear-cut dysplastic features in their neutrophils. The model learning was only based on the single-cell annotation of the neutrophils from pMDS, without human-generated morphological features as input. The trained neutrophil classifier achieved an overall accuracy of 94%, with a sensitivity and specificity of 0.95 and 0.94, respectively. On a patient-level, the model correctly identified 91 out of the 94 samples, with a sensitivity and specificity of 0.98 and 0.96, respectively, and AUC of 0.999. In npMDS, 43 out of the 44 samples were correctly identified. Our study demonstrates the potential of AI-based models to improve the efficiency of MDS diagnostics. Our model runs on standard CPUs, offering an accessible solution that can be integrated into existing clinical workflows and potentially reduces the dependence on specialized morphologists and genomic analysis from bone marrow punctures.

**Supplementary Information:**

The online version contains supplementary material available at 10.1007/s00277-025-06533-5.

## Introduction

Myelodysplastic syndromes/neoplasms (MDS) are a group of blood disorders defined by ineffective hematopoiesis, resulting in peripheral cytopenia and an elevated risk of progression to acute myeloid leukemia (AML) [1, 2]. Whereas the status of molecular markers must be assessed in MDS to determine the optimal treatment strategy, the initial diagnosis relies on the morphological analysis of the peripheral blood smear or bone marrow aspirate under a microscope.

However, identifying subtle morphological changes requires expert knowledge and is considered a major human bottleneck for efficient analysis. Automated cell image devices in hematology units can generate and categorize thousands of single-cell images within minutes [3]. Despite these advances, the assessment still depends on the morphologist’s opinion, characterized by low sensitivity and inter-observer variability. This is particularly relevant in MDS, where the degree of dysplasia can vary from very mild to extremely pronounced. The disease spectrum can range from mild forms with low impact on quality of life to more aggressive forms that rapidly progress into acute leukemia. Accordingly, reported incidence rates vary widely from 3.3 to 4.9 per 100’000 in the US [4]to as high as 75–162 for the population above 65 [4, 5]. This discrepancy is not only due to the historically missing awareness but rather because the diagnosis of MDS at the initial stage is often overlooked. Genomic markers such as del(5q), *TP53* and *SF3B1* mutations are present only in about half of MDS patients [6] and this testing is cost-intense, has long turnaround times, and is not always definitive [7, 8].

Recent advances in computer vision (artificial intelligence) have shown promise in improving diagnostic accuracy and efficiency across fields, such as radiology, histopathology and hematology [8]. Recently, the potential benefits of implementing computer vision solutions as first-line diagnostics in resource-limited settings have been discussed, in particular, to rule out patients who do not require advanced diagnostic follow-up [9–11]. In hematology, computer vision has been used to automate tasks such as detecting, counting and subtyping blood cells on peripheral blood smears (PBS) [12, 13]. However, its application to direct disease diagnosis remains limited. As recently reviewed, most AI studies focus on leukemia, with only one single study addressing MDS detection [14, 15].

In this study, we approached diagnosing MDS with computer vision to enhance diagnostic accuracy by standardizing the assessment of cellular morphology. We developed an AI model capable of diagnosing MDS using automatically acquired single-cell images from PBS without relying on human-generated morphological features for its training.

## Methods

### Data cohort and ethical approval

Blood smears were prepared from routine EDTA blood samples using the SP50 system (Sysmex). On all samples, automated digital cell classification was performed using Di-60 (Sysmex) and CellaVision Peripheral Blood Application Software (Lund, Sweden). MDS patients’ blood samples were digitized at first diagnosis. The diagnosis was confirmed by bone marrow aspiration and two hematologists using the WHO 2016 classification, as well as by genomic analyses [2]. A total of 84 samples from 84 MDS patients were retrospectively included, with a 2.4:1 male: female ratio. Patients were between 32 and 88 years old at the time of sampling (median age 69). The control cohort consisted of 60 putatively healthy blood samples without known history of hematological disorder. Exclusion criteria consisted of being under 18 years of age, or not giving general consent to research. The study was conducted according to the regulations of the local ethics committee (BASEC number 2021 − 00111) and the Declaration of Helsinki.

### Sample acquisition, digitization and annotation

**Fig. 1:**
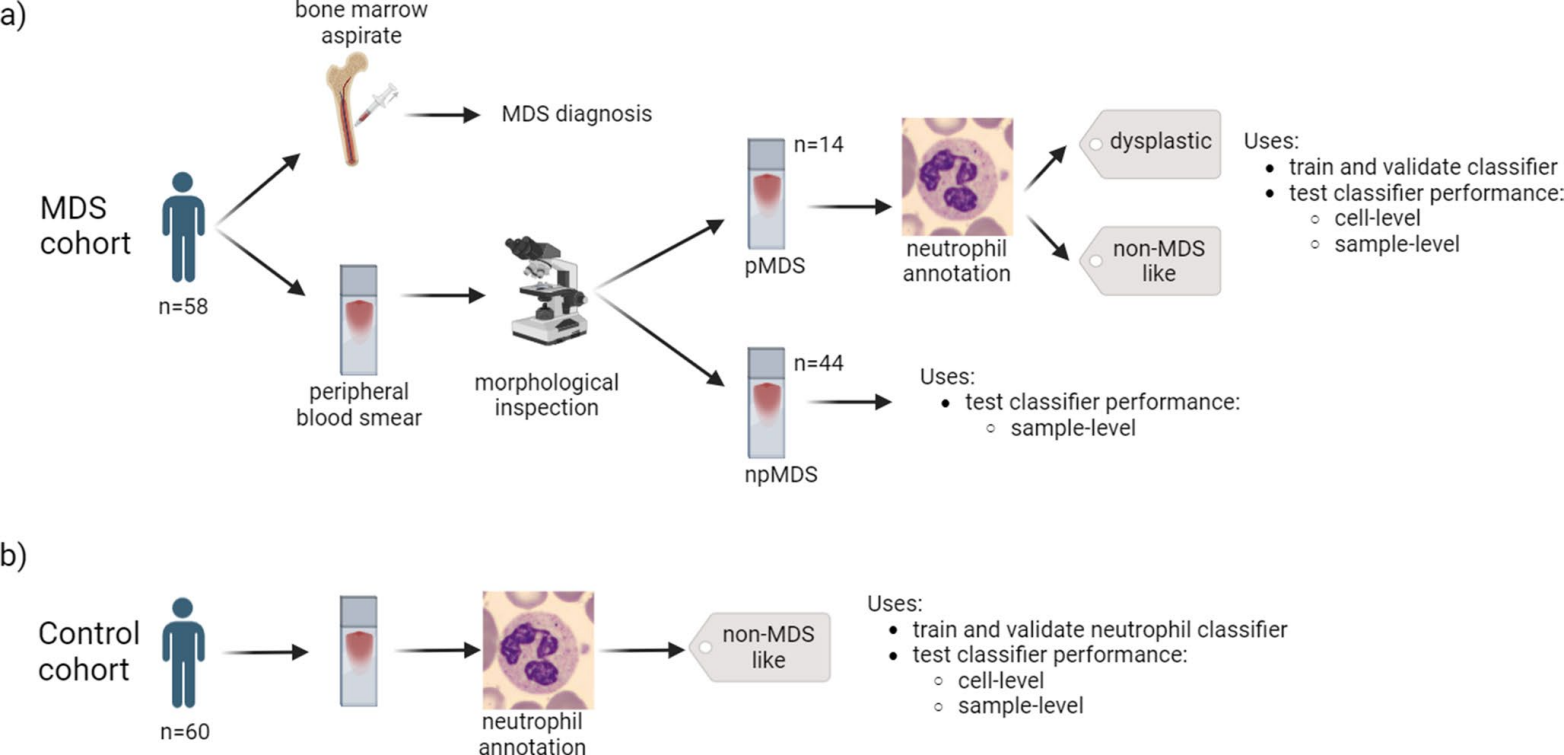
Sample cohort and peripheral blood smear data. (**a**) MDS patients were diagnosed by bone marrow aspirate according to WHO guidelines. Their peripheral blood smears were inspected by an expert morphologist and annotated as either prominent MDS (pMDS) or non-prominent MDS (npMDS). pMDS samples were annotated at the single-neutrophil level by an experienced hematologist and used for neutrophil classifier training and validation, followed by testing at the single-cell level. npMDS samples were combined with the pMDS test set to evaluate model performance at the sample-level. (**b**) Putatively healthy donors provided peripheral blood smears, which yielded neutrophils for classifier training and validation, as well as model performance testing at the cell-level and sample-level. Samples and their constitutive neutrophils were confined to a single datasplit (training, validation, or testing) for the entire workflow, ensuring that model evaluation was performed on never-before-seen samples

From each blood smear, a median of 521 (up to 7100) images were taken and stored in JPEG format. This resulted in a pool of 96’727 images, each classified for cell type by the CellaVision system. From the 84 MDS patient samples, 14 were selected to train and validate the neutrophil classifier, as an expert morphologist considered these blood smears to exhibit strong signs of morphological dysplasia (prominent MDS or “pMDS”, Fig. 1). This subset yielded 5,234 segmented neutrophil and band neutrophil images, which were individually annotated as either: (i) “dysplastic”, i.e. morphologically prominent dysplasia showing segmented or non-segmented Pseudo-Pelger-Huët anomaly and abnormal granulation (hypogranular or agranular cytoplasm) or (ii) “non-MDS like”, i.e. few or no clear-cut dysplastic changes. Annotations were performed in Labelbox by an expert morphologist (Labelbox, San Francisco). For the remaining 70 MDS samples, 26 were excluded due to failed sampling or fewer than 10 neutrophils imaged. The remaining 44 “non-prominent MDS” samples, having been validated by bone marrow aspiration but not exhibiting prominent morphological dysplasia in the blood smear, were reserved for patient-level validation of the neutrophil classifier. For the control cohort, all 19’075 segmented and band neutrophils were automatically labeled as “non-MDS like” (Supplementary Table 1).

### Dataset split

The neutrophil classifier was trained on 5804 neutrophils (*n* = 2969 non-MDS like cells, *n* = 2835 dysplastic). The model was optimized using a validation set (*n* = 1016 non-MDS like, *n* = 807 dysplastic), and final model performance was quantified with a hold-out test set (*n* = 1763 non-MDS like, *n* = 404 dysplastic). For additional information about dataset split and neutrophil classifier evaluation, see Supplementary Information.

### Cell segmentation and image pre-processing

To segment neutrophils from their background, a custom U-Net++ architecture was selected to balance pixel-level accuracy with the possibility of lightweight inference on CPU [16]. The segmentation model was trained on an open-source Blood Cells Image Dataset, comprising 17’092 blood cells imaged on the CellaVision DM96 system [17]. Ground truth masks were automatically generated by deploying a pre-trained Segment Anything Model SAM 2 (Meta) in zero-shot mode, followed by a post-processing filtering protocol consisting of erosions, dilations, and object filtering. See Supplementary Information for U-Net characteristics and image standardization performed.

### Neutrophil classifier training and sample-level prediction

To classify neutrophils as either non-MDS like or dysplastic, an EfficientNetV2 architecture with a binary classifier head was selected for its balance of computational efficiency and performance [18]. Once the preprocessing protocol was selected, the final classifier was trained by fine-tuning a model pre-trained on ImageNet. Samples with more than 10% dysplastic neutrophils were classified as MDS-positive.

## Results

**Fig. 2:**
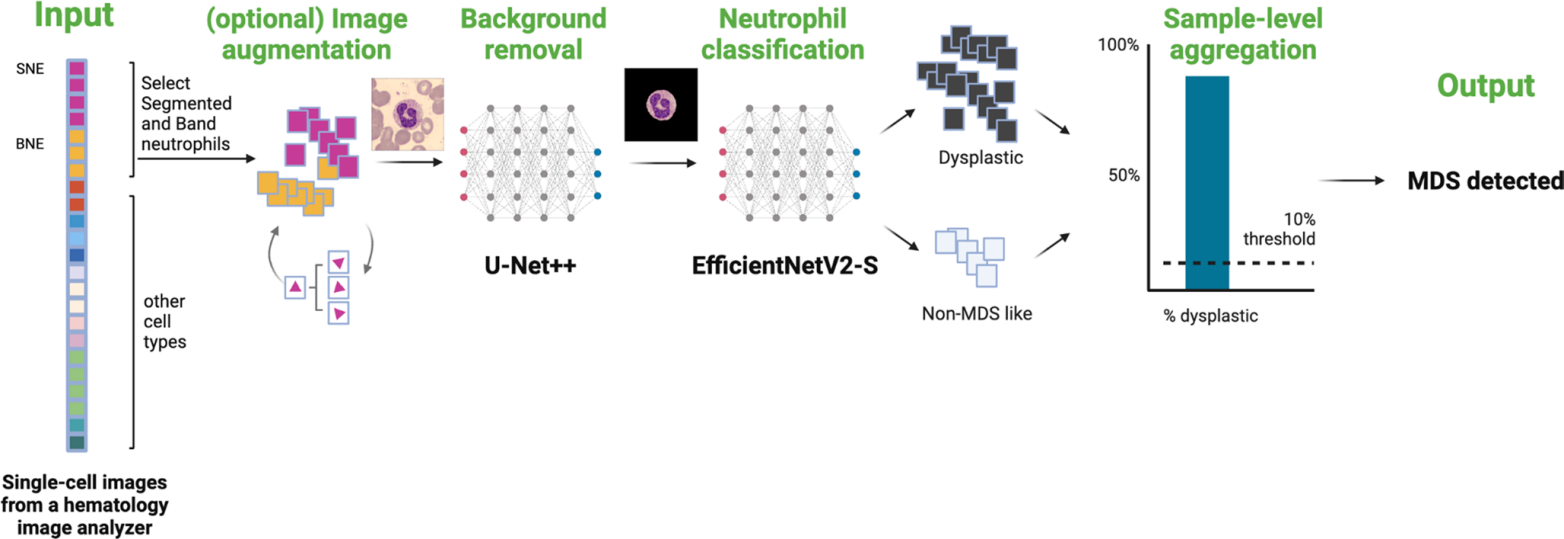
Overview of the MDS detection pipeline. Neutrophil single-cell images were subjected to augmentation, background removal, and classification according to the presence of dysplastic morphology indicating MDS. For the final MDS classification on a patient level, the percentage of neutrophils predicted as dysplastic was compared to a predefined threshold

**Fig. 3:**
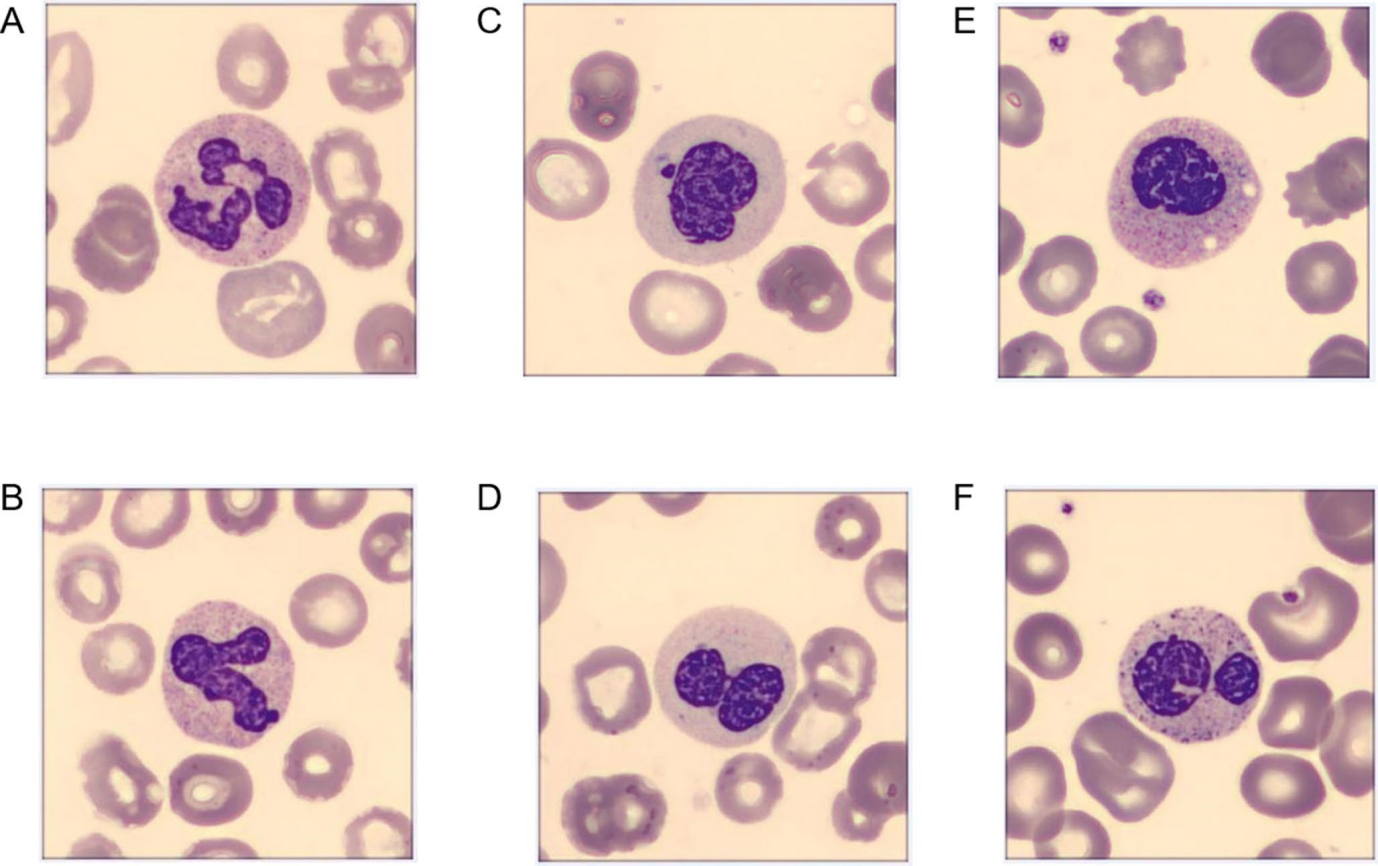
Representative images of segmented neutrophils (SNE) from patients with MDS showing no prominent dysplastic (**A-B**) or dysplastic features (with prominent non-segmented and segmented Pseudo-Pelger-Huët forms, C-F, and agranular cytoplasm **C** and **D**

The starting point of this study was to develop a model capable of diagnosing MDS using routine imaging of PBS (Fig. 1). Figure 2 outlines the model training pipeline, from single-cell image generation to sample-level prediction. Neutrophil morphology in MDS patients ranged from non-prominent to heavily dysplastic forms (Fig. 3). In 14 pMDS patients, 4’443 single-cell images of neutrophils were annotated (median of 334 neutrophils per patient), with 4046 and 397 cells labelled as dysplastic and non-MDS like (i.e., no visual sign of dysplastic features), respectively.

### Single-cell segmentation to remove background context

**Fig. 4:**
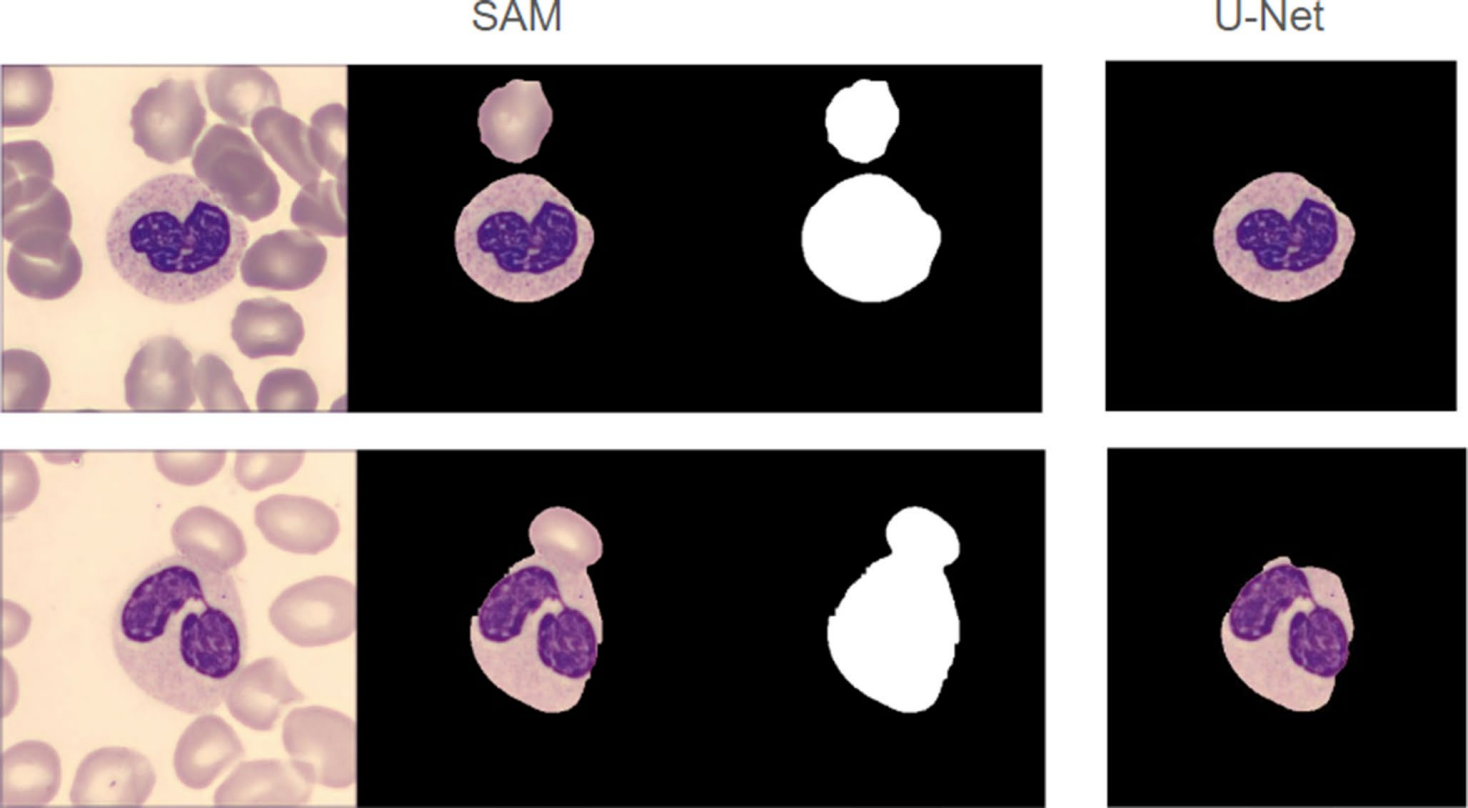
SAM and U-Net mask applications on representative images

To ensure that the neutrophil classifier identified features related to sub-cellular morphology and to limit the possibility of context clues confounding the results, a semantic segmentation model was trained to identify the central cell in the image patch and remove background pixels. We trained a custom U-Net++ on a dataset consisting of single-cell masks generated by a pretrained SAM 2 ViT-h model and evaluated it by comparing the masks from 1507 neutrophil image patches with the ones generated from SAM. This strategy leveraged the power of a 636 million parameter foundation model to generate thousands of masks in a zero-shot manner. The U-Net recapitulated the much more computationally intensive SAM V2 with a Jaccard score of 98.5% (95% CI [0.983–0.986]) (Fig. 4), as well as being lightweight and CPU-compatible.

### Training and evaluation of the neutrophil classifier for the detection of dysplasia

**Fig. 5:**
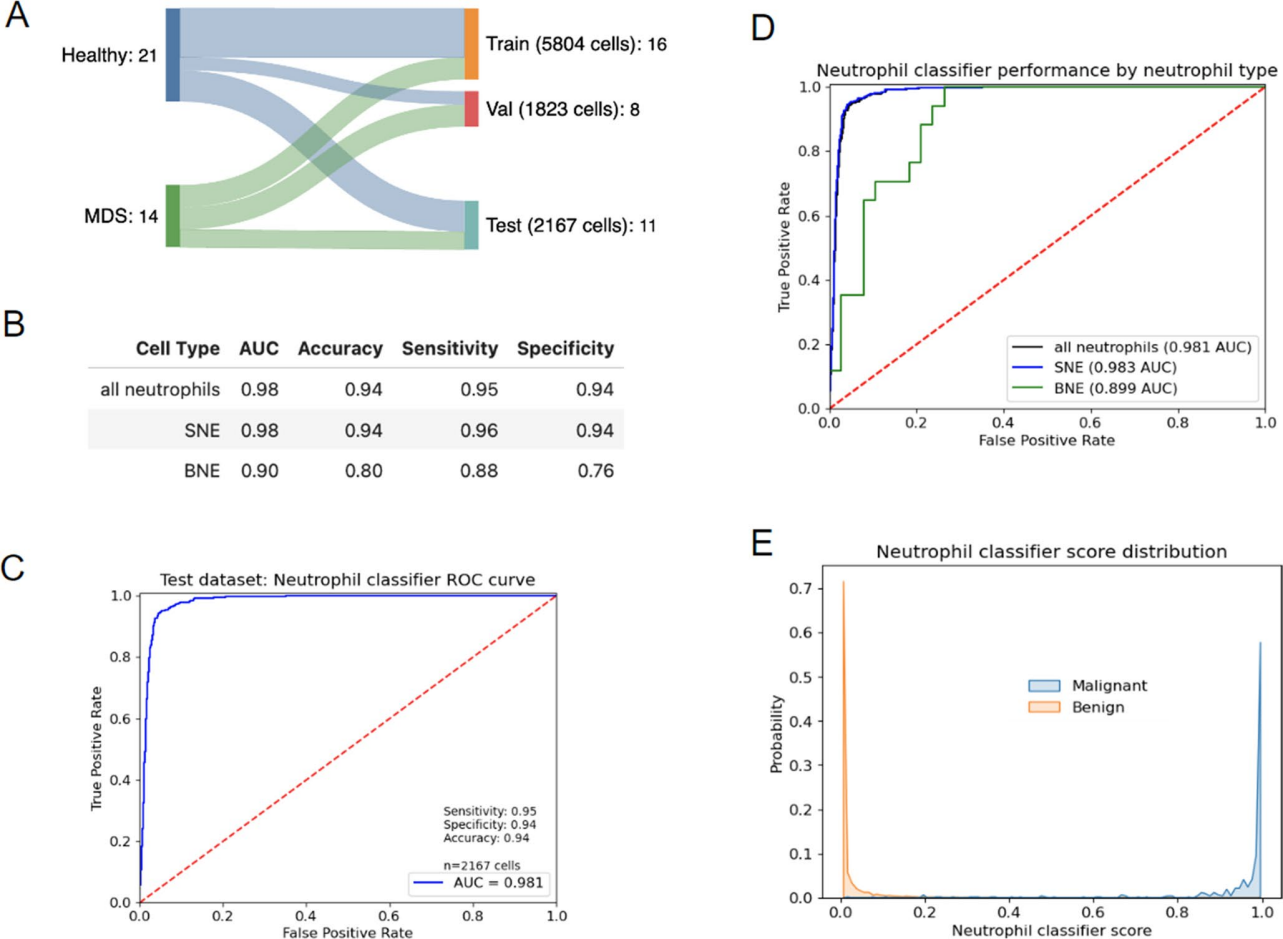
Neutrophil classifier. (**A**) Data split overview of single cells from either prominent MDS patients or putatively healthy individuals. (**B**) Summary of neutrophil classifier performance metrics for all neutrophils, segmented neutrophils (SNE), and band neutrophils (BNE), as evaluated on the test data split of 2,167 neutrophils. (**C**) Receiver Operating Curve (ROC) for model predicting dysplasia in segmented and band neutrophils. (**D**) ROC curves for neutrophil classifier by cell type: SNE, BNE, and all neutrophils. (**E**) Distribution of scores from neutrophil classifier, as applied to the test data split of 2,167 segmented and band neutrophils from 11 donors. Score distributions are separated according to the expert annotation of that cell’s morphology as “dysplastic” or “non-MDS like”

Using the background-filtered images of segmented and band neutrophils, we trained a classifier to detect dysplastic neutrophil morphology by using images from patients with pMDS that had previously been annotated by expert hematologists. We divided all annotated single-cell images into three independent cohorts of training, validation, and test sets before model training (see Fig. 5a, Supplementary Table 2). We used six combinations of standardization and data augmentation protocols to train six experimental classifiers with EfficientNetV2-S architectures and binary output heads on a subset of 5,652 image patches from the training data split. We selected Model 3, with a brightness jitter at 10% of the maximum range, as the most performant in terms of F1-score and sensitivity, for final model training (see Supplementary Table 3).

The final classifier was applied to the hold-out test set comprising 1,763 non-MDS like and 404 dysplastic cells and achieved a sensitivity and specificity of 0.95 and 0.94, respectively, and an overall accuracy of 0.94 (Fig. 5). Model accuracy for segmented neutrophils was similarly high (sensitivity: 0.96, specificity: 0.94). For banded neutrophils, the classifier exhibited an accuracy of 0.80 and a sensitivity and specificity of 0.88 and 0.76, respectively. The model generated 111 false-positive cell predictions and 19 false negatives, with the segmented neutrophil population accounting for 102 of the false positives and 17 of the false negatives. Band neutrophils were more likely than segmented neutrophils to be misclassified, as confirmed with a Z-test of two proportions (*p* < 0.0001). When evaluating samples from the control cohort, 82% of the misclassifications occurred in a single sample, all being segmented neutrophils. For MDS samples, 93.7% of misclassifications occurred in two of the four samples, both of which were subdiagnosed as MDS with excess of blasts 2 (Supplementary Fig. 1).

### Application of the neutrophil classifier as a patient-level classifier for non-prominent MDS detection

**Fig. 6:**
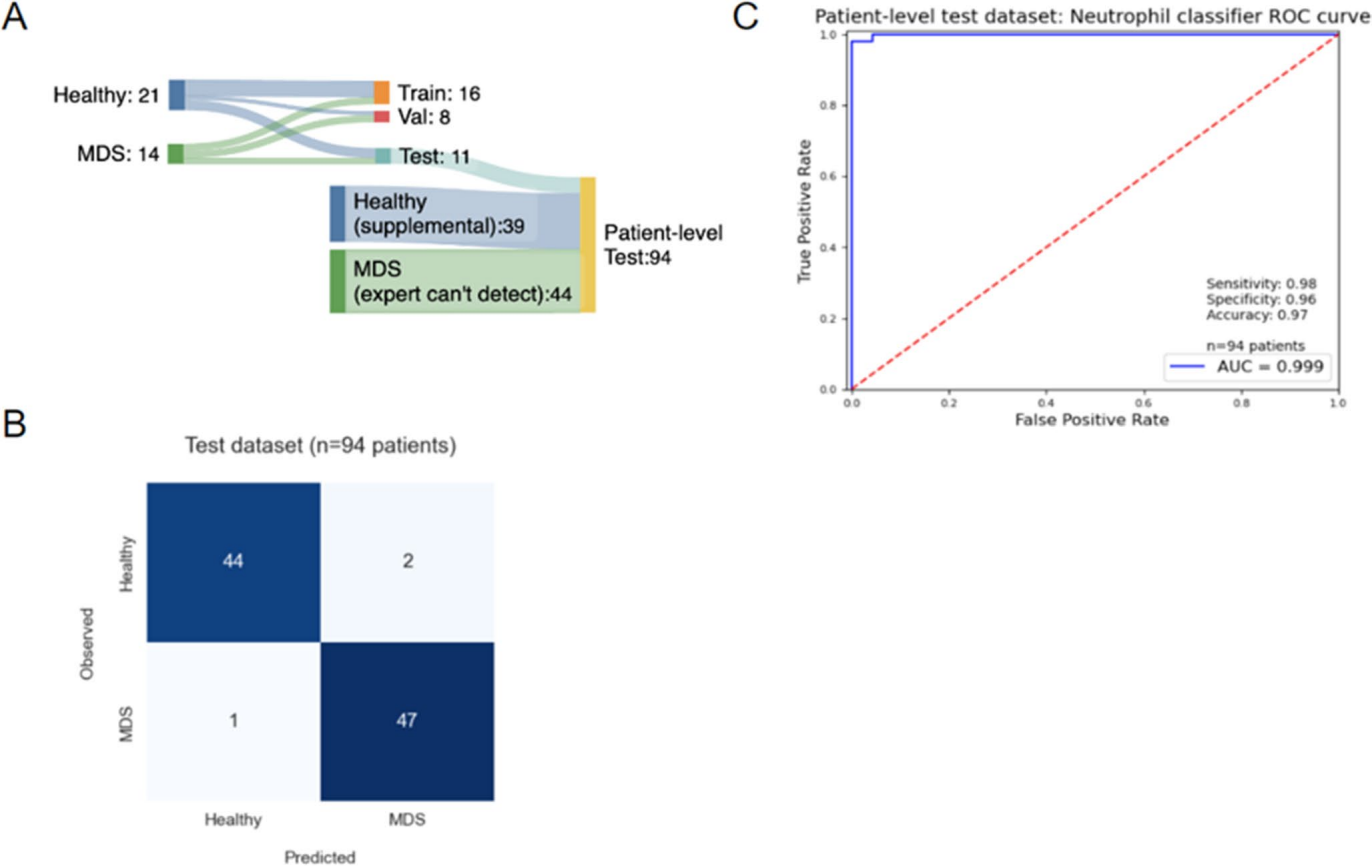
MDS patient classifier. (**A**) Data split overview of patient samples from the MDS and control cohorts. (**B**) Confusion matrix for classification on the test dataset, with a 10% dysplastic neutrophil threshold. (**C**) Receiver Operating Curve for classifier predicting MDS disease

Next, we wanted to determine if the trained neutrophil classifier could detect MDS in patients whose neutrophils showed no morphologically prominent detectable signs of dysplasia (npMDS). For this purpose, we assembled a test cohort of 94 individuals comprising 44 patients with non-prominent MDS (Fig. 6). To evaluate the single-cell classifier’s performance on a patient level, we calculated the fraction of neutrophils predicted to be dysplastic by the model and applied a threshold of 10% dysplasia to indicate MDS positivity, mimicking the cutoff used by morphological experts in the clinic and recommended by WHO guidelines [2].

To generate a comprehensive model for the detection of MDS at the whole-sample level, a logistic regression was fit using the percentage of neutrophils as dysplastic along with several additional cell population features related to the presence of distinct cell subpopulations, such as giant thrombocytes or lymphocyte level. However, the percentage of dysplastic neutrophils was a highly performant feature, with 99% of instances being predicted correctly, resulting in quasi-complete separation and a failure of the logistic regression to shed light on the discriminative power of the additional features.

Application of our model and the threshold of 10% to the 35,744 neutrophils from the 94 blood smears led to a correct detection of MDS in 91 out of the 94 samples, with one false negative and two false positive results, leading to a sensitivity and specificity of 0.98 and 0.96, respectively, an accuracy of 0.97 and an area under the curve of 0.999 (Fig. 6). The one false negative originated from the npMDS cohort and showed a neutrophil positivity rate (7.7%) close to the chosen cutoff of 10%, whereas the two false-positive patient samples had more than 14% neutrophils predicted as dysplastic (Fig. 6b).

**Fig. 7:**
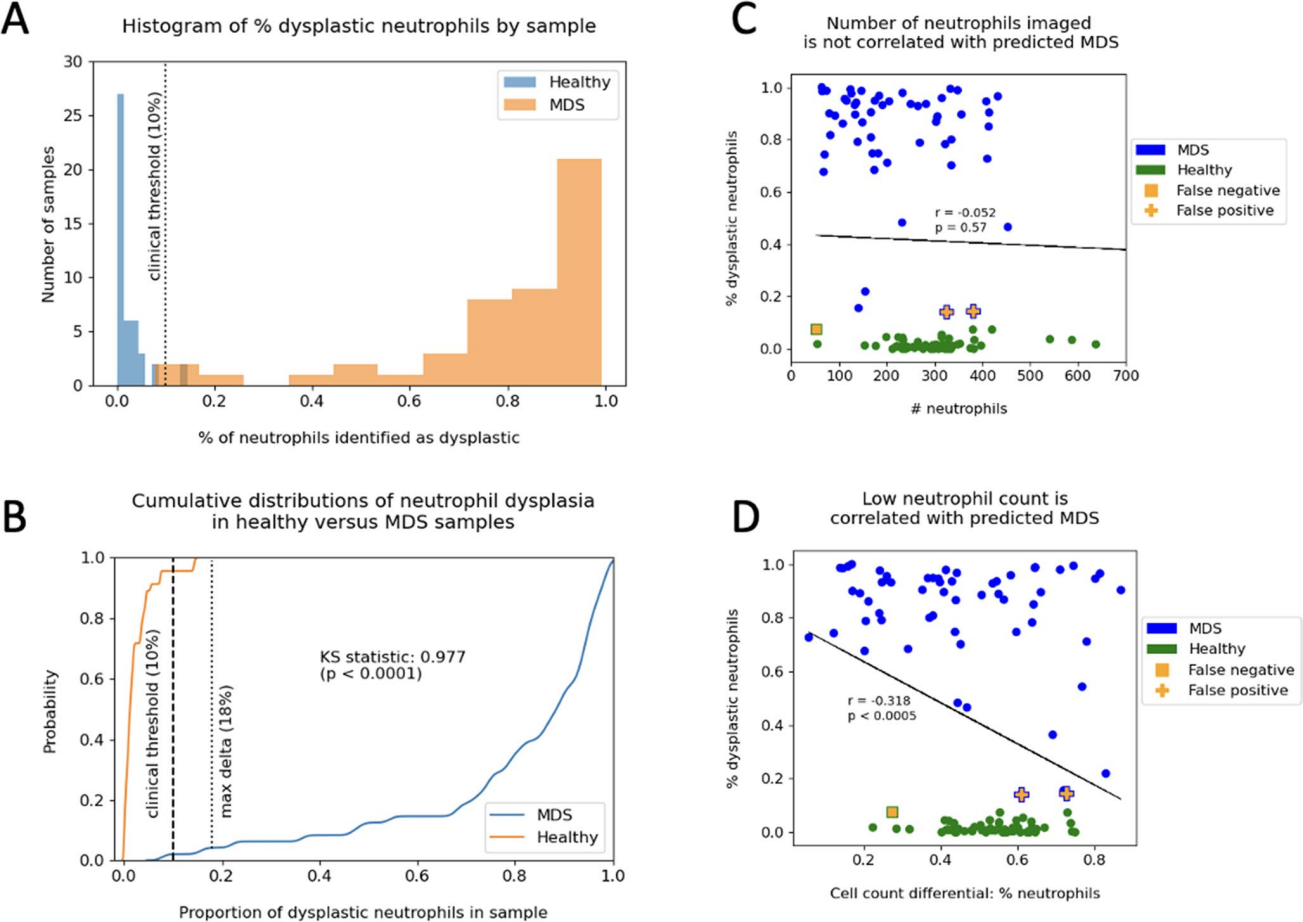
Percentage of dysplastic neutrophils per sample. (**A**) MDS and control samples in the hold-out test set could be discriminated by calculating the percentage of neutrophils predicted as dysplastic. (**B**) The clinical threshold of 10% dysplasia was empirically validated by comparing the cumulative distribution functions of the rate of dysplasia in MDS versus control samples. A Kolmogorov-Smirnov test of two samples revealed a maximum delta of 0.977 at 18% dysplasia, with *p* < 0.0001. (**C**) The probability of a sample being predicted as having MDS was not a function of the number of neutrophils evaluated (*p* = 0.57). (D) A low neutrophil count in the sample was correlated with predicted MDS (*p* < 0.0005), validating clinical expectations for low neutrophil counts in MDS patients

To evaluate if the frequency of dysplasia is a valuable feature for MDS detection, we examined the rate of neutrophil dysplasia in control versus MDS samples (Fig. 7). This examination validated the clinical threshold of 10% dysplasia as indicative of MDS: a Kolmogorov-Smirnov test of two populations indicated that the rate of dysplasia in control samples is significantly lower than that of MDS samples, with a maximum delta of 0.977 at 18% dysplasia (Fig. 7b). Next, we examined whether evaluating a too-small number of neutrophils might influence the overall sample-level prediction (Fig. 7c). We found no statistical correlation between the absolute number of neutrophils examined and the predicted patient status, with an r-value of −0.052 (*p* = 0.57). In contrast, a linear least-squares regression revealed that the differential cell count of neutrophils in the sample was highly correlated to the predicted patient status, with an r-value of −0.318 (*p* < 0.0005). This finding is supported clinically, as patients with MDS typically present with cytopenia.

## Discussion

Our study highlights the advantages of combining lightweight and performant computer vision architectures with state-of-the-art laboratory infrastructure to provide precise, rapid, and cost-effective MDS diagnostics that are scalable for clinical use. The current diagnostic workflow relies on highly specialized morphological expertise to identify dysplastic features in the cells of blood or bone marrow smears and usually involves genomic analyses such as NGS or karyotyping [19]. Despite rapid advances in multi-omics technologies, the vital importance of highly accurate morphological evaluation to classify hematological malignancies is still anchored in the current WHO classification of hematolymphoid tumors [20]. The cutoff for dysplasia to be classified as pathological is also a matter of debate, as real-world data from healthy bone marrow donors also show a surprisingly high prevalence of dysplasia-related signs [21]. Furthermore, data registry trials with central cytological evaluation by expert cytologists provide deep insights into patterns of dysplasia in MDS patients [22]. In routine clinical practice however, centralized expert cytological reviews are not routinely available, highlighting the necessity for reproducible point-of-care assays to detect dysplasia/MDS.

Previous studies have shown that computer vision models can distinguish MDS from anemia by analyzing peripheral blood smears images [15, 23]. These studies relied on human-annotated features or the availability of hardware-intensive resources. In our study, we forced our model to use only the neutrophil morphology without relying on contextual cues such as red blood cell density. Second, we achieved this focus on cell morphology without relying on the use of any human-annotated features by leveraging the use of an open-source dataset and foundation model for its training. Third, we leveraged deep neural networks to extract de novo features, enabling the neutrophil classifier to detect MDS-positive samples even in the absence of prominent dysplasia. And finally, our solution lowers the resource requirements for integration into a diagnostic lab by requiring only CPU-based hardware and integrating directly with a hematology image analyzer. It is essential that the resulting application be efficiently run on standard CPUs and not rely on expensive GPUs that are typically unavailable in resource-constrained hematology laboratories [24, 25].

For cell segmentation, we aimed to balance the generalizability of a foundation model with the lightweight performance of a custom-trained model, acknowledging that the latter historically outperforms the former on medical imaging data [26, 27]. To generate an annotated training dataset rapidly and without utilizing significant human resources, we leveraged SAM 2 as a foundation model to generate zero-shot segmentation masks on an open-source Blood Cells Image Dataset [17]. The choice to train our segmentation model on an open-source dataset was designed to promote generalizability across institutions. While exciting new methods are emerging to perform resource-efficient transfer learning on foundation models, these models still rely on costly GPU infrastructure and compute resources for inference [28]. Despite its significantly smaller parameter count (9 million to SAM’s 636 million), our U-Net achieved near-parity with SAM 2, with a Jaccard score of 98.5% and a reduction in over-segmentation of nearby cells.

The neutrophil classifier was trained on restricted datasplits, ensuring that the model learned from a high-quality set of neutrophils exhibiting prominent morphological signs of dysplasia, while providing a generous test set to measure the model’s ability to detect more subtle signals in non-prominent MDS samples [27]. Consistent with previous reports, we observed that model accuracy in this imaging domain is highly sensitive to augmentation strategies [29]. Image rotation and patch-level normalization improved model accuracy by 8.5%, and the careful application of brightness jitter improved accuracy by another 3.5–96.4% before any other model optimization.

The neutrophil classifier correctly classified 2037 of the 2167 neutrophil cells in the cell-level test set, achieving an overall accuracy of 0.94 (AUC 0.98). In MDS samples, false-positive predictions dominated the misclassifications. Further analysis would be required to understand if these false positives might not already comprise dysplastic features that are not visible to the human eye but are detected by the higher sensitivity of the model. Furthermore, 94% of the misclassified cells in MDS patients originated from two samples, both with the subdiagnosis of MDS with excess blasts. Only one of these two MDS samples showed a high misclassification rate of more than 25% of the cells; in that sample, 80% of the misclassifications were false positive predictions, i.e., cells that had been annotated as morphologically non-dysplastic (non-MDS like), but classified by the model as dysplastic.

Our classifier correctly identified 91 out of the 94 patients in the test-set. The three misclassifications included two false-positives and one false-negative, with the latter showing a neutrophil positivity rate of 7.7%, close to the selected threshold of 10%. This predetermined threshold of 10% was chosen based on clinical practice and WHO guidelines. Although used in clinical routine, we are aware that this threshold is arbitrarily chosen and that there are reports questioning its validity [21]. Further, it is also controversially discussed whether there is a pathognomonic sign of dysplasia or a typical pattern of a combination of different signs of dysplasia, as the number of dysplastic signs per patient can be relatively low [22]. While a single cutoff is practical, introducing an “intermediate” category could allow for additional follow-up testing or clinical review for borderline cases. Additionally, our model relies exclusively on dysplasia to detect MDS, resulting in potential false-negative predictions for cases with only excess blasts or aplastic blood cell count. Nevertheless, our findings suggest that neutrophil dysplasia in neutrophils may be universally present across all MDS subtypes, and this may be an underestimated feature, as most patients in the study were correctly classified based on dysplastic features in their neutrophils. To reflect real-word diagnostic variability, we included both morphologically prominent (pMDS) and non-prominent (npMDS) cases in our dataset. This decision was critical to ensure that our dataset captures the full spectrum of variability, including intrapatient heterogeneity with normal, npMDS and pMDS neutrophils coexisting in the same sample. Such intra-patient heterogeneity poses a unique diagnostic challenge and underscores the need for robust algorithms to detect and interpret mixed morphologies. Our approach aligns with current literature emphasizing the necessity of validating diagnostic tools on datasets that reflect real-world heterogeneity.

We are aware that our study has limitations. The dataset was derived from a single-center cohort, which may limit generalizability. Furthermore, the study did not evaluate the model’s performance in detecting other hematological diseases. Future work will expand the dataset to include a variety of conditions, thereby validating the model’s broader applicability. We are convinced that computer vision-based applications such as the one presented in this study can reshape diagnostic workflows by making diagnostics more efficient and less expensive and lowering the access barrier to testing. Future efforts could focus on refining thresholds to minimize false negatives in first-line screening while maintaining a balance to avoid unnecessary follow-ups.

Our study demonstrates that deep learning for morphological image analysis can offer practical, cost-effective, and scalable solutions for modern diagnostics without requiring significant investments in specialized hardware, human-annotated data for model development, or extensive changes in diagnostic workflows. By leveraging CPU-based with existing hematology infrastructure, it achieves significant efficiency gains while ensuring robust diagnostic accuracy.

## Supplementary Information

Below is the link to the electronic supplementary material.

ESM 1 (DOCX 2.38 MB)

## Data Availability

The data used for this study are subject to national data protection laws and are available from the corresponding author upon reasonable request. Access to the data requires authorization by the ethical committee and the execution of a Data Sharing Agreement. Interested researchers can contact the corresponding author for further information and to initiate the request process.
