# Supplementary material for: AI-based detection of neutrophil dysplasia: an accessible and sensitive model for MDS diagnosis from peripheral blood
Source: Ann Hematol. 2025 Aug 19;104(9):4477–86. doi: 10.1007/s00277-025-06533-5 (PMC12552336; doi:10.1007/s00277-025-06533-5)
Supplement: Supplementary file 1 — (DOCX 2.38 MB) [file 277_2025_6533_MOESM1_ESM.docx]

**Supplementary Information/Data**

**U-Net and image standardization**

The U-Net, initialized with weights from an ImageNet-trained model, was trained on a training and validation data split of 80:20, with image transformations consisting of flips, flops, and rotations. Standardization and augmentation strategies were tested against one another to determine the optimal protocol. For image standardization, an RGB mean and standard deviation was determined for each image patch, for all image patches in a sample, or from the entire training datasplit. In two of the protocols, a jitter was added on brightness, contrast, saturation, and/or hue at 10% or 20% of the sample value range (Supplementary Table 1). After augmentation, the cell segmentation mask was applied to remove the background.

**Datset split and neutrophil classifier performance evaluation**

Each sample (stemming from a unique patient) was confined to a single dataset split for the entirety of the study: training (n=5 MDS patients, n=11 control), validation (n=5 MDS, n=3 control), and hold-out test sets (n=4 MDS, n=7 control). Samples were allocated to maximize the diversity of MDS diagnosis codes (subtypes) in each dataset split.

The training, validation and test set for the neutrophil classifier comprised 5804 (composed of 2969 non-MDS like and 2835 dysplastic cells), 1823 (1016, 807) and 2167 (1763, 404) neutrophils, respectively (Figure 5a.)

To evaluate the neutrophil classifier performance at the patient level, the test set from above was augmented with the npMDS cohort and previously unused control samples for a total of n=43 npMDS and n=39 control, the former of which could not be annotated at the single-cell level due to subtlety of morphologic changes. For model integrity, all test datasets were firewalled and not accessed until after all models were finalized.

**Hardware/platform characteristics**

The final model pipeline was implemented on an n1-standard-4 instance from Google Cloud Platform, with 4 vCPUs and a total of 15GB of memory.

**Evaluation of the Neutrophil Classifier Performance**

For the experiments, a subset of 5,652 image patches from the training data split was used. Each model’s performance was evaluated on a 1’600 image subset of the validation split for accuracy, F1-score, sensitivity, specificity, and recall. Two models stood out: Experiment 3 with the best accuracy, F1-score, and sensitivity, and Experiment 6 with the best specificity and precision. Performance in terms of F1-score and sensitivity were considered the most important factors in prioritizing the detection of dysplastic neutrophils while optimizing for the most performant part of the ROC curve in order to not miss MDS patients who could further progress. Under these criteria, model 3 (see Supplementary Table 2: Experiment 3), characterized by image patch-based standardization with brightness jitter at 10% of the maximum range, was considered the most performant. As expected, the least performant model was generated without standardization or data augmentation. Consequently, for final model training, a preprocessing step of image patch-based standardization with only brightness jitter at 10% was used.


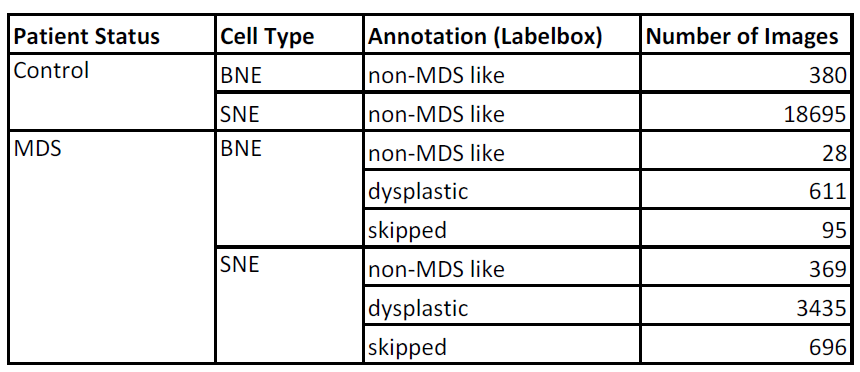


**Supplementary Table 1**. From putatively healthy donors, 19,075 segmented and band neutrophils were automatically labeled as having a non-dysplastic morphology. From MDS patients, a subset of 5,262 segmented neutrophil and band neutrophil images were further classified into two groups: i) showing clear dysplastic changes (Pelgeroid changes) with extreme neutrophil nucleus changes and/or agranular cytoplasm or ii) absence of such clear changes, with further classification options for imaging artifacts obscuring the morphology. Images with artifacts or with indeterminate status were skipped.


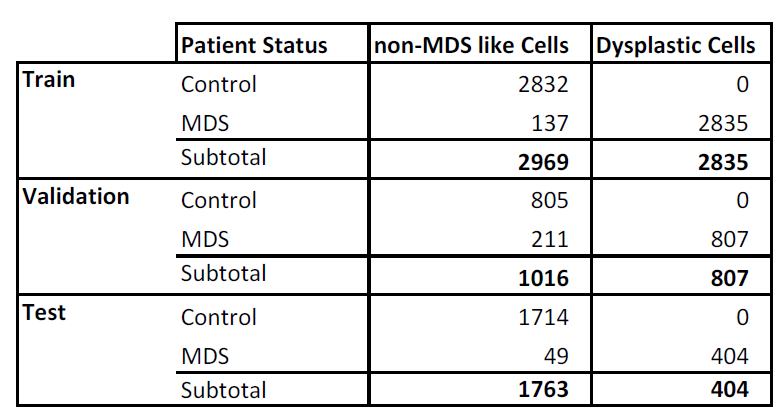


**Supplementary Table 2**: Annotated neutrophils used for the single-cell classifier, originating from 14 patients with pMDS and 21 putatively healthy individuals.


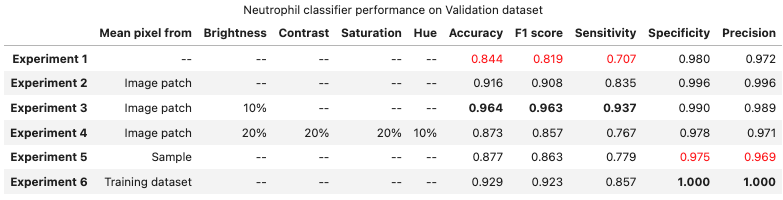


**Supplementary Table 3**. Neutrophil classifier performance is sensitive to image normalization and augmentation methods during model training. Models were trained on images normalized according to mean pixel values calculated from the individual image patch, the entire patient sample, or the whole training set. Additionally, image augmentation was performed by applying a jitter to the image patch brightness, contrast, saturation, and/or hue, as a % of the maximum range in the training dataset. Accuracy, F1-score, Sensitivity, Specificity, and Precision of the model were recorded for each of the 6 classifiers, using the validation data split to benchmark model performance.


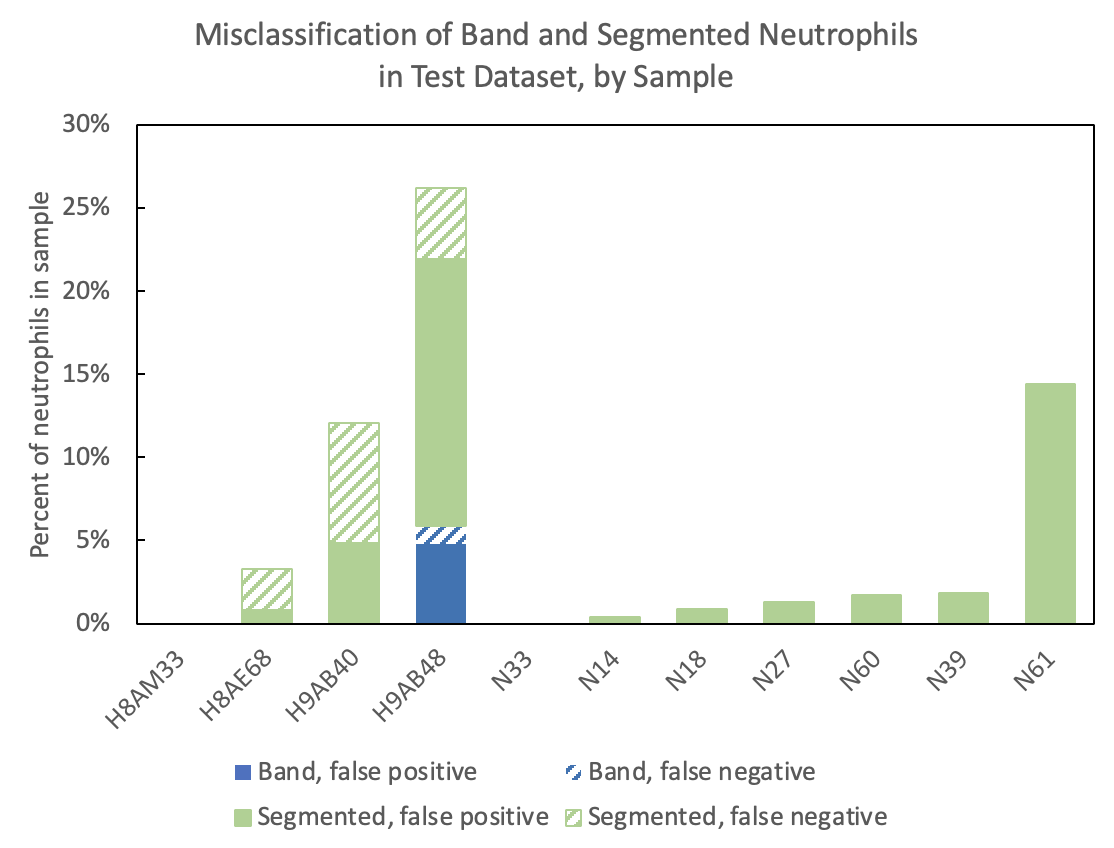


**Supplementary Figure 1.** Categorization of misclassifications by the neutrophil classifier, by sample and neutrophil type.
